# Pilot screening of potential matrikines resulting from collagen breakages through ionizing radiation

**DOI:** 10.1007/s00411-024-01086-z

**Published:** 2024-08-08

**Authors:** Juliette Montanari, Lucas Schwob, Aurélie Marie-Brasset, Claire Vinatier, Charlotte Lepleux, Rodolphe Antoine, Jérôme Guicheux, Jean-Christophe Poully, François Chevalier

**Affiliations:** 1 UMR6252 CIMAP, CEA - CNRS - ENSICAEN - Université de Caen Normandie, Caen, 14000 France; 2 Deutsches Elektronen-Synchrotron DESY, Notkestr. 85, 22607 Hamburg, Germany; 3 Nantes Université, CHU Nantes, INSERM, Regenerative Medicine and Skeleton, RMeS, UMR 1229, Oniris, Nantes, F-44000 France; 4 Institut Lumière Matière, University of Lyon, Université Claude Bernard Lyon 1, CNRS, Lyon, F-69622 France; 5 UMR6252 CIMAP, CEA-CNRS-ENSICAEN-Université de Caen Normandie, Bd Henri Becquerel - BP 55027, CAEN Cedex 05, F-14076 France

**Keywords:** Collagen matrikine, Irradiation, Bystander effect, Chondrocytes, Peptide fragmentation

## Abstract

Little is known regarding radiation-induced matrikines and the possible degradation of extracellular matrix following therapeutic irradiation. The goal of this study was to determine if irradiation can cut collagen proteins at specific sites, inducing potentially biologically active peptides against cartilage cells. Chondrocytes cultured as 3D models were evaluated for extracellular matrix production. Bystander molecules were analyzed in vitro in the conditioned medium of X-irradiated chondrocytes. Preferential breakage sites were analyzed in collagen polypeptide by mass spectrometry and resulting peptides were tested against chondrocytes. 3D models of chondrocytes displayed a light extracellular matrix able to maintain the structure. Irradiated and bystander chondrocytes showed a surprising radiation sensitivity at low doses, characteristic of the presence of bystander factors, particularly following 0.1 Gy. The glycine-proline peptidic bond was observed as a preferential cleavage site and a possible weakness of the collagen polypeptide after irradiation. From the 46 collagen peptides analyzed against chondrocytes culture, 20 peptides induced a reduction of viability and 5 peptides induced an increase of viability at the highest concentration between 0.1 and 1 µg/ml. We conclude that irradiation promoted a site-specific degradation of collagen. The potentially resulting peptides induce negative or positive regulations of chondrocyte growth. Taken together, these results suggest that ionizing radiation causes a degradation of cartilage proteins, leading to a functional unbalance of cartilage homeostasis after exposure, contributing to cartilage dysfunction.

**Supplementary Information:**

The online version contains supplementary material available at 10.1007/s00411-024-01086-z.

## Introduction

The term “matrikines” refers to the degradation products of the extracellular matrix (ECM), mainly peptides, able to induce a biological response in the cells of this micro-environment (Maquart et al. 2005). Matrikines were initially designated to be products of ECM proteins resulting from the action of cellular matrix metalloproteinases (MMPs). This cellular mechanism was suggested as a new process capable of activating several intracellular signaling pathways, resulting in the modulation of numerous cell functions, such as cell adhesion, proliferation, migration, apoptosis or matrix degradation (Siméon et al. 1999; Jariwala et al. 2022). In cartilage tissues, collagen is degraded by matrix metalloproteinases such as MMP-13, leading to a common joint disease, osteoarthritis (Troeberg and Nagase 2012). In addition to enzymatic degradation, exposure to radiation and reactive oxygen species (ROS) can fragment ECM proteins (Vartio 1989; Watson et al. 2014). Medical irradiation such as radiotherapy involves high doses of X-rays targeting cancer cells, but it can reduce conjunctive tissue functionality too (Saintigny et al. 2015). Local inflammation and fibrosis are major side effects of radiotherapy, due to the loss of the equilibrium between ECM production and degradation which usually maintains tissue homeostasis (Bonnans et al. 2014).

It is therefore essential to know, understand, and quantify the effects induced by the irradiation of this omnipresent matrix during radiotherapy, and in particular the possible formation of matrikines. These molecules can act on cancer cells, but also diffuse outside the irradiated zone and act as stress signals for healthy cells, or even induce their senescence (Blokland et al. 2020). In addition, these peptides could contribute to radiation-induced diseases such as osteoarthritis, the latter usually being due to the degradation of cartilage through aging (Willey et al. 2013; Little et al. 2019).

We previously demonstrated such a bystander effect following irradiation with X-rays and carbon ions of chondrosarcoma cells. Chondrosarcomas are cartilage tumors in which the proportion of ECM is significant (Chevalier et al. 2019). The conditioned medium of irradiated chondrosarcoma cells showed a significant activity on non-irradiated chondrocytes. The nature of the cellular response remained uncertain and several effectors were proposed to be involved in this bystander effect, including TNF-alpha, stress granules and reactive oxygen species (Lepleux et al. 2019; Tudor et al. 2021; Gilbert et al. 2022).

In this study, we examined whether chondrocytes synthesize ECM in vitro and how irradiation by carbon ions or X-rays promotes ECM degradation. Using a medium transfer protocol, we demonstrate the presence of a bystander effect between irradiated and non-irradiated chondrocytes at low radiation doses. A highly preferential breakage site in collagen due to direct radiation effects was deduced from mass spectrometry experiments: the glycine-proline (Gly-Pro) peptide bond. Then, we selected 46 peptides resulting from Gly-Pro cleavage of collagen, and tested their toxicity in vitro against chondrocytes. We finally proposed a list of collagen peptides with contrasted cellular activity.

## Materials and methods

### 2D cell culture

A chondrocyte cell line, T/C28-A2 (from Prof. Mary B. Goldring, Hospital for Special Surgery, Weill Medical College of Cornell University, New York, NY, USA) was cultured in RPMI-1640 medium (Roswell Park Institute Medium 1640, Sigma-Aldrich), supplemented with 10% fetal calf serum, 2 mM L-glutamine and 1% antibiotics (Penicillin-Streptomycin solution, Sigma-Aldrich). All experiments were performed in humidified atmosphere with 5% CO_2_ at 37 °C, in a Heracell™ 150i incubator. The cells are periodically tested for mycoplasma contamination.

### 3D pellets cell culture

From T/C-28A2 cells cultured in 2D, 1 million cells were detached and centrifuged for 10 min (4 °C, 600 rpm). Then, the supernatant was removed and the pellet was detached with 500 µL of 3D culture medium and centrifuged again for 10 min at low speed (4 °C, 600 rpm). Tubes (15 ml, conical) containing each pellet were placed in the incubator (37 °C, 5% CO_2_) in order to allow the formation of “small balls” called “pellets” until 15 days. The medium for 3D cell culture is composed with DMEM (DMEM - high glucose D6429, Sigma Aldrich), 5% Fetal calf serum, 10 ng/mL BMP-2 (Bone morphogenetic protein 2, NBP1-19751, Novus), 0.1 mM insulin (CC-4147, Lonza), 0.1 mM ascorbic acid (CC-4147, Lonza), 2 mM glutamine and 0.1% hydrocortisone (CC-4147, Lonza). In order to characterize our 3D cellular models by different staining processes, paraffin inclusions are made. These experiments were carried out on the histology platform (PFSC3M, INSERM, UMRS1229-RMeS) in Nantes, France. Each 3D model were fixed in 4% paraformaldehyde, embedded in paraffin and cut in 5 μm thick sections using microtome. Hematoxylin-eosin-safran (HES), Alcian Blue and Masson’s Trichome staining have been performed to visualize cells and matrices, as previously described (Merceron et al. 2010).

### Irradiation of cells

X-ray irradiations were performed with a X-RAD Smart 225Cx irradiator (Precision X-ray Inc.) using a tube tension of 225 kV, dedicated to preclinical research, with a lower energy compared to clinical radiotherapy as previously described (Chevalier et al. 2019; Lepleux et al. 2019). The energy spectrum of the photons provided by the irradiator used in our studies has not been measured. Le Deroff and colleagues (Le Deroff et al. 2019) have simulated this spectrum and validated their results by comparing the experimental and simulated absorption and dose in aluminium. In the conditions relevant to our experiments, the spectrum is composed of tungsten lines superimposed on a continuous bremsstrahlung emission with maximum and mean photon energies of 225 and 84.8 keV. All irradiations were performed at the medium position of the sample holder, in case of low doses irradiations (0.05 to 0.2 Gy), an intensity of 1 mA corresponding to a dose rate of 0.2 Gy/min was selected, and in case of doses between 0.5 and 8 Gy an intensity of 10 mA corresponding to a dose rate of 2 Gy/min was selected.

### Clonogenic assays of irradiated T/C-28a2 cells

This method was used to screen the sensitivity of cells to different radiation doses. For this approach, cells were irradiated at low density in 25 cm^2^ flasks. A sham irradiated control was performed to evaluate the plating efficiency, it represents the 0 Gy sham control. After an incubation period of 10 days, colonies were stained with a crystal violet solution (0.3% w/v crystal violet in 20% v/v ethanol). Colonies composed of at least 50 cells were counted visually with a stereomicroscope. The results (*N* = 3) are expressed as a percentage of the control un-irradiated cells (mean +/- SD).

### Clonogenic assay of bystander T/C-28a2 cells

This experiment was used to estimate the bystander effect against cell survival with a “medium transfer” protocol from irradiated cells to non-irradiated cells. For this protocol, irradiated T/C-28a2 cells were plated in T25 cm^2^ flasks at confluency. The conditioned medium was collected 24 h after irradiation, centrifuged (2000 g) and transferred on flasks of the same size (T25 cm^2^) containing non-irradiated bystander T/C-28a2 cells at low density. After an incubation period of 10 days, colonies were stained with a crystal violet solution (0.3% w/v crystal violet in 20% v/v ethanol). Colonies composed of at least 50 cells were counted visually with a stereomicroscope. The results (*N* = 3) are expressed as a percentage of the control un-irradiated cells (mean +/- SD).

### Irradiation and fragmentation of peptides in the gas phase

**Fig. 1:**
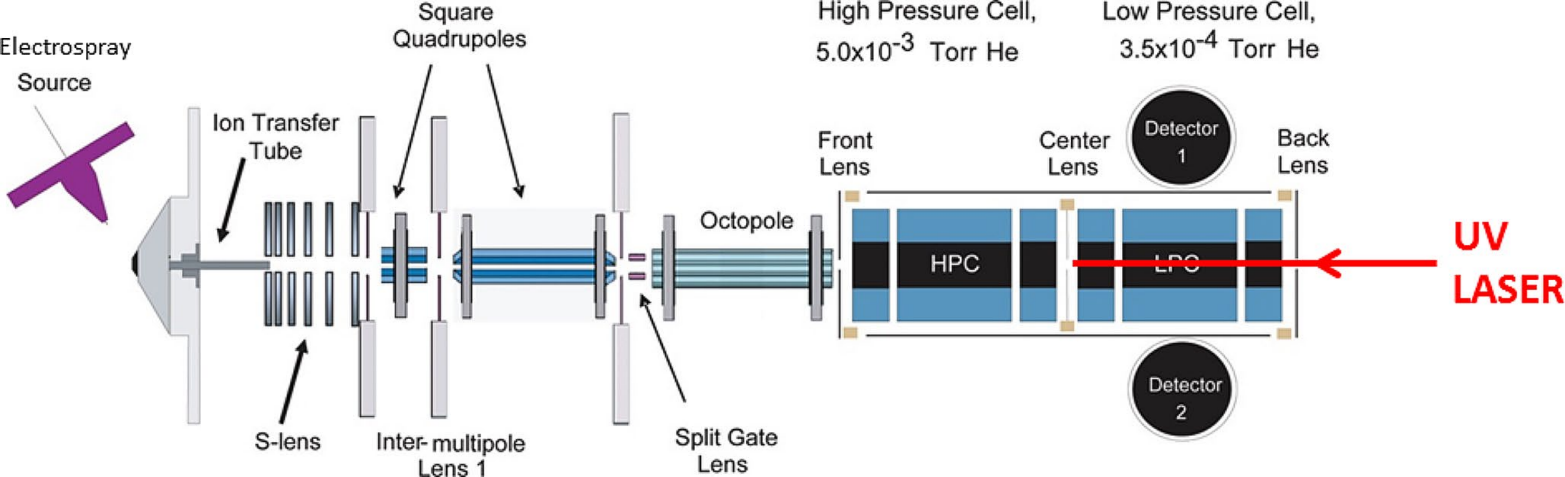
Experimental set-up used to record the mass spectra of the cationic products of collagen peptide ions upon collision-induced dissociation (CID) or UV photoabsorption at 220 nm (see Fig. 4). The peptide ions are produced by an electrospray ionization source, guided through several RF devices, trapped in a high-pressure cell where CID is performed, and the products are transferred to the low-pressure cell to be mass-analyzed. UV irradiation is carried out in the high-pressure cell; adapted with permission from (Pekar Second et al. 2009)

Synthetic peptides PK26 (average mass = 2274.4 Da; sequence PGG-PPG-PKG-NSG-EPG-APG-SKG-DTG-AK) and PK26-Hyp (average mass = 2322.4 Da; sequence PGG-POG-PKG-NSG-EOG-AOG-SKG-DTG-AK where O is hydroxyproline) were purchased from ProteoGenix (France). Samples of over 95% purity were used with no further purification and were prepared in 50:50 (volume ratio) water/methanol solutions at a 50 µM peptide concentration with 1% of formic acid for protonated peptides, or 1% of ammonium hydroxide for deprotonated peptides. The experimental setup used at the Institut Lumière Matière (Lyon, France) for collision-induced dissociation (CID) and UV photofragmentation of peptides has been described in details previously (Antoine and Dugourd 2011; Bellina et al. 2013). In brief, a dual linear ion trap mass spectrometer (LTQ Velos, Thermo Fisher Scientific, San Jose, CA) was modified to allow the UV laser beam to interact with the peptide ions, by positioning a fused silica window (25.4 mm diameter, 3 mm thick) at the back end of the instrument, and drilling circular openings in the trapping electrodes (Fig. 1). The peptide cations or anions were brought in the gas phase thanks to an electrospray ionization source in positive or negative mode, respectively. The peptide ion with the charge state of interest was accumulated and isolated in the first ion trap prior to interacting during 100–500 ms with either helium gas at 10^− 3^ mbar and 10–30% normalized collision energy for CID experiments, or with a UV laser nanosecond pulse at 220 nm for photofragmentation. The UV laser (220 nm) is a Horizon OPO pumped by the third harmonic of a Surelite I Nd: YAG laser (Continuum, Santa Clara, CA), with power 3 mW, repetition rate 10 Hz, and pulse width 5 ns. A mechanical shutter, synchronized with the mass spectrometer, was used to control the irradiation time. Then, the precursor ions remaining in the trap and the product ions from collision with helium or UV photoabsorption were transferred in the second ion trap, where mass analysis was performed. The mass spectra presented in this article are the result of averaging over a few minutes.

### Peptide synthesis

All peptides used in the MTT cytotoxicity tests were synthesized by solid-phase techniques and provided by Proteogenix (Schiltigheim, France) with over 95% purity. The peptide powders were not further purified.

### MTT cytotoxicity test

MTT assay was carried out to assess the chemical sensitivity of chondrocyte cells to collagen peptides. In brief, media supplemented with peptides were used to culture the cells in 96-well plates for 48 h. Afterward, 10 µL MTT solution (Gibco, USA) was added to each well and incubated at 37 ˚C for an additional 4 h, and then 100 mL dimethyl sulfoxide (DMSO) was used to dissolve the crystal. The optical density (OD) at 490 nm was determined by a micro-plate reader (Thermo Fisher USA), then we obtained the inhibition rate of cells, as a mean of 4 repetitions of two independent experiments. The design formula was as follows: cell inhibition rate (%) = 1−(experimental group OD value/normal group OD value) × 100%.

### Data analysis and statistical tests

Peptide toxicity tests were analyzed using the Orange data mining software version 3.36.1. The heatmap visualization tool was used with a clustering / ordering of peptides. Data are clustered by similarity with hierarchical clustering on Euclidean distances and with average linkage. It additionally maximizes the sum of similarities of adjacent elements. The statistical analysis of clonogenic assays was performed using the statistical module of the Origin software (V 6.0), by a t-test (two populations) with an independent type and a 0.05 significant level. Data sets were considered as significantly different when *p* < 0.05 (*). Similarity analysis of peptides was performed with COBALT tool (NCBI) (Papadopoulos and Agarwala 2007). It does progressive multiple alignment of protein sequences. The alignment is aided by a collection of pairwise constraints derived from conserved domain database, protein motif database, and local sequence similarity using RPS-BLAST, BLASTP, and PHI-BLAST, respectively.

## Results and discussion

### 3D chondrocyte models produce an extracellular matrix containing collagen

As a first step, a characterization of our cellular model was performed, in order to define and quantify the capacity of the cells to synthesize ECM. A 3D-pellets culture model, based on a centrifugation of the cells and then a culture under non-adherent condition of the resulting pellet, was selected for this characterization. The immortalized human chondrocyte cell line T/C-28A2, previously described to be able to produce ECM was used for this study (Goldring et al. 1994). Following an inclusion and paraffin section of our model after 15 days of culture, we carried out three types of staining: coloring with HES, with Alcian Blue, and with Masson Trichrome in order to characterize our 3D models.

**Fig. 2:**
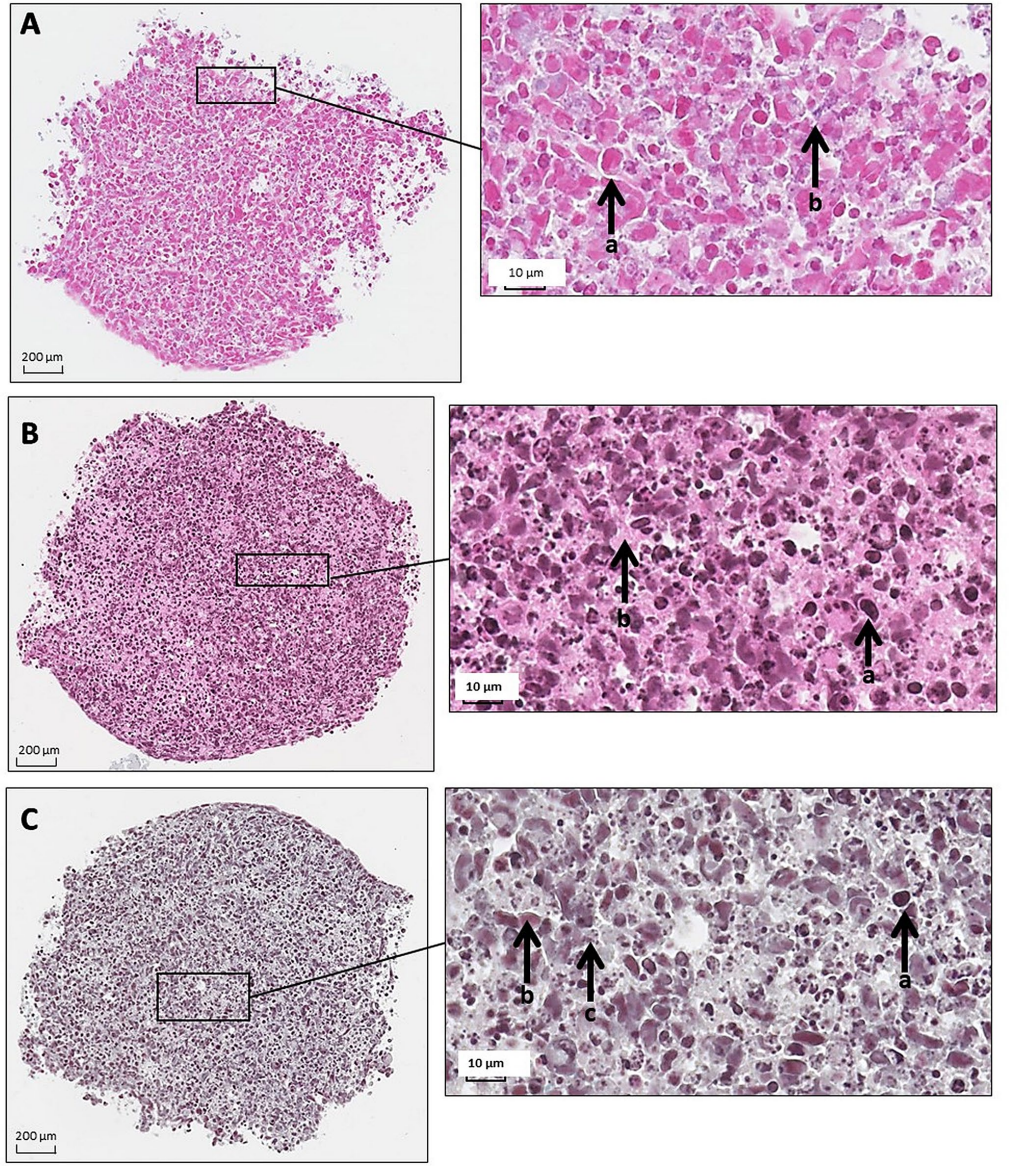
Histological section of T/C-28a2 pellet at day 15. (Nanozoom2.0 HT – Hamamatsu, 800x lens). Enlarged part: 80x zoom via NDP View2 software. **A**: HES staining, with cell nuclei in blue-black (b) and cell cytoplasm in pink (a); **B**: Alcian Blue staining, with glycosaminoglycane (GAG) chains in blue (b) and cell nuclei in red (a); **C**: Masson’s Trichome staining, with cell nuclei in blue-black (a), collagen in light green, cytoplasm in pink-red (b, c)

HES staining (Fig. 2A) highlights the nuclei and cytoplasm of cells as well as the connective tissue of the extracellular matrix. The nuclei are colored blue-black, the cytoplasm is pink and the collagen is orange-yellow. According to Fig. 2A (enlarged part), we can clearly distinguish the cells and their nucleus (blue-black) (b) as well as their cytoplasm (pink) (a). The Alcian Blue coloring (Fig. 2B) allows us to highlight the Glycosaminoglycans (GAGs). GAG chains can be covalently linked to protein to form proteoglycans which are essential components of the ECM. This staining therefore allows us to highlight the capacity of cells to produce ECM. The nuclei are colored red, the GAGs blue. According to Fig. 2B (enlarged part), we notice numerous round and red markings (a) which show the presence of numerous nuclei. However, a slight blue coloring is difficult to observe, indicating a low quantity of GAGs present (b). Masson’s Trichrome (Fig. 2C) allows us to highlight the connective tissue (collagen). This staining allows the nuclei to be colored blue-black or even brown (a), the collagen and mucus in light green and the cytoplasm in pink-red (b, c). According to Fig. 2C, we observe many nuclei within the section associated with some collagen protein of the ECM.

The characterization of the pellets by these three stainings allowed us to visualize the high cellularity of this model composed of T/C-28a2 cells and also a light ECM (little connective tissue, proteoglycans and collagen). However, even if the ECM is present in small quantities, it allows the maintenance of the 3D structure.

### A high radiation-sensitivity of chondrocytes at low dose

As a second step, we analyzed the survival response of cells after irradiation. T/C-28A2 cells were irradiated and kept in culture for 10 days with potential fragments of the ECM and the survival rate was estimated using a clonogenic assay.

**Fig. 3:**
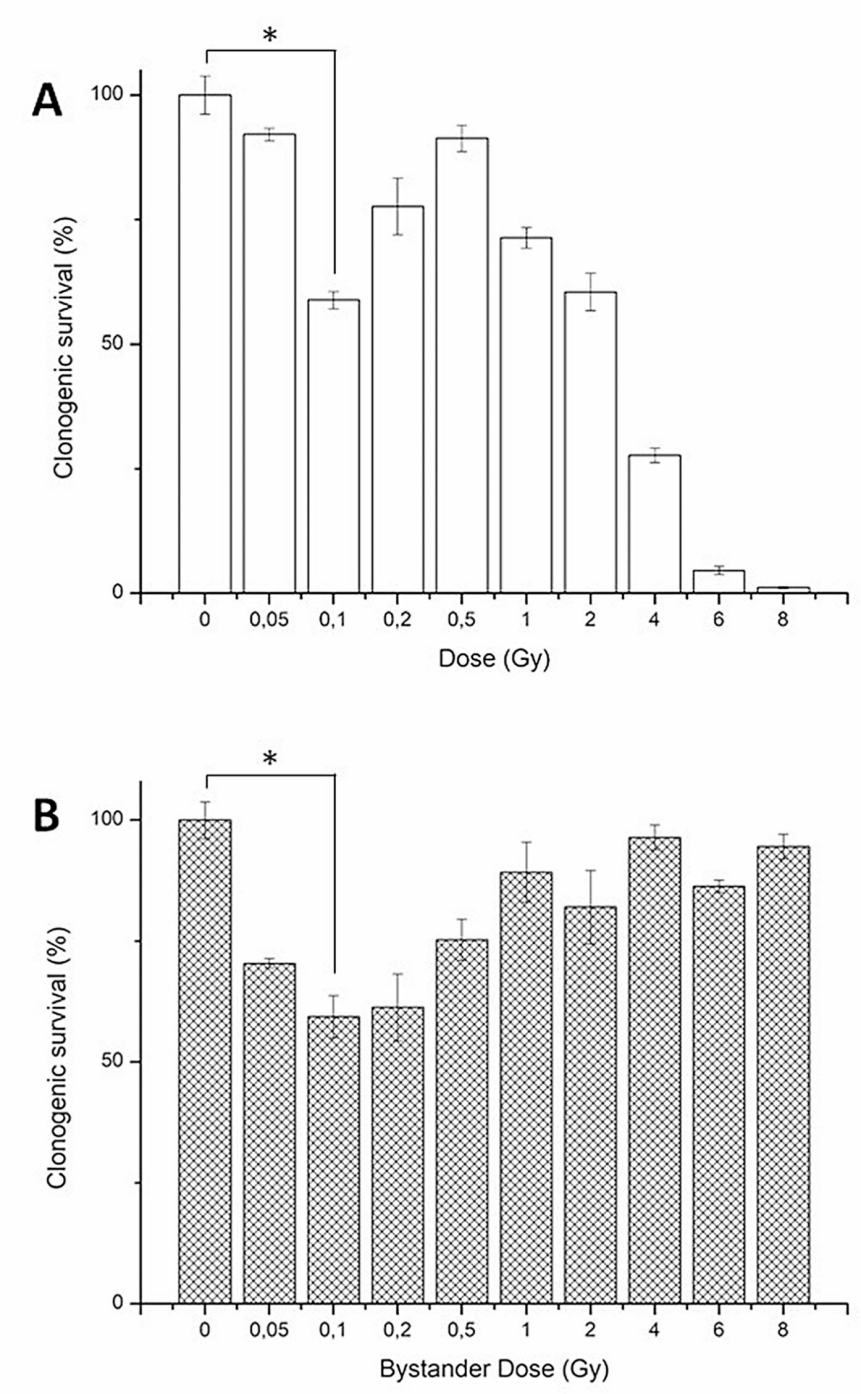
Clonogenic survival (%) of (**A**): x-ray irradiated chondrocytes according to irradiation doses; (**B**): bystander chondrocytes, receiving the conditioned media of x-ray irradiated chondrocytes at different doses (bystander dose), from three independent experiment (mean +/- SD)

For this, the cells were irradiated by X-rays at different doses from 0.05 to 8 Gy and the survival rate was estimated using the non-irradiated cells as control (100%). Thus, as can be seen in Fig. 3A, we observe a dose-dependent reduction in the clonogenic survival of chondrocyte cells after X-ray irradiation between 0.5 and 8 Gy, as expected. But a surprising hypersensitivity at low doses was observed, especially at 0.1 Gy with a significant reduction of about 40% of the survival rate.

To better understand the cellular mechanism of such hypersensitivity at low dose, we performed a medium transfer protocol on non-irradiated cells. Briefly, 24 h after irradiation, the conditioned medium of irradiated cells was removed and transferred on non-irradiated chondrocytes and the survival rate was estimated using a clonogenic assay, in the same way as with irradiated cells. Thus, if a factor from the medium was responsible for this hypersensitivity at low dose, it would be observed on non-irradiated cells. Indeed, a significant reduction of the survival rate was observed on bystander cells receiving the conditioned medium from irradiated cells (Fig. 3B), but only at low doses between 0.05 and 0.2 Gy.

Such phenomenon was previously observed using chondrosarcoma cells (SW1353 cell line) irradiated with the same conditions (Lepleux et al. 2019). Irradiated chondrosarcoma cells were described to produce in vitro bystander factors inducing a 36% survival rate on T/C-28a2 cells after a transfer of medium. These bystander factors were produced at a maximum amount when chondrosarcoma cells were irradiated with 0.1 Gy of X-rays. A deep characterization of the bystander factors was performed using ELISA tests and proteomic studies. Several factors were proposed to contribute for this bystander effect, including cytokines and other stress factors such as stress granules (Lepleux et al. 2019; Tudor et al. 2021; Gilbert et al. 2022), but only a fraction of the bystander effect could be related to such secreted factors. As proposed previously, other molecules, produced by irradiation could be also implicated (Chevalier et al. 2014), including peptide fragments of the ECM proteins (Sherratt et al. 2010; Watson et al. 2014; McCabe et al. 2020). In addition, we cannot rule out the possibility that irradiated proteins from the culture medium (such as serum albumin) could produce diluted peptides too.

It is interesting to note that chondrosarcoma cells are able to produce bystander factors following irradiation, but these cancer cells are not sensitive to these factors (Wakatsuki et al. 2012), in contrast to chondrocytes that are able both to produce and receive bystander factors following irradiation. This is the reason why a radiation sensitive effect was observed both with irradiated and bystander cells (Fig. 3). As proposed previously, ECM fragments could be involved in such bystander effects, but these fragments are largely unknown, especially regarding collagen. Under these culture conditions, with a monolayer of cells cultured in 2D, T/C-28a2 display a low level of extracellular matrix proteins (Finger et al. 2003). But this artificial 2D cell culturing allows a homogeneous irradiation of all cells and extracellular matrix proteins, all at the same dose and same level. Under the close vicinity between the cell and the plastic of the flasks, protein and peptide concentration can be higher, inducing potential biological effects.

Therefore, with the goal to identify such factors, we irradiated isolated collagen-related peptides and looked for a preferential cleavage sites using mass spectrometry analysis of the ionic fragments obtained.

### Direct effects of ionization or electronic excitation: the Gly-Pro peptide bond is a preferential cleavage site

In order to identify putative preferential cleavage sites in collagen resulting from the interaction with particles of different natures and energies, in this and previous work we have performed irradiation of peptides in the gas phase, and analyzed the product ions by means of mass spectrometry. Our method allows unambiguously determining which bonds are cleaved in the peptide.

### Previous results

In previously published studies, we have deeply investigated the response of collagen mimetic peptides upon ionizing photons as well as carbon ions at the Bragg-peak kinetic energy (around 1 MeV/u). Collagen mimetic peptides were originally designed for their ability to form good-quality crystals for X-ray diffraction experiments, allowing studies of the influence of the amino acid sequence (and thus of mutations) on the triple helix structure typical of collagen (Shoulders and Raines 2009). For our studies, we have chosen the well-known (PPG)_10_ and (POG)_10_ peptides that have a glycine every three residues and a high proline content, like collagen. Moreover, (POG)_10_ contains hydroxyproline residues, present in unusually high abundance in collagen, enhancing its stability and resulting from a post-translational modification of proline residues in Yyy position in the XxxYyyGly repeat (Bhattacharjee and Bansal 2005). We have found that these protonated peptides irradiated in the gas phase undergo ionization but also fragmentation of the peptide backbone. These processes are due to absorption of one VUV or soft X-ray photon or to interaction between one carbon ion and molecular electrons, followed by deposition of excess vibrational energy in the peptides (Schwob et al. 2017b; Lalande et al. 2018a). The mass spectrum measured for carbon ion irradiation is very similar to the one for 150 eV photons, showing that the same peptidic fragments are created after cleavage of backbone bonds. Interestingly, the Gly – Pro peptide bonds are cleaved with very high probability compared to other bonds in the peptide, independently of the photon energy or the nature of the particle (photon or carbon ion). We have also probed the influence of a molecular environment on the radiation-induced processes in collagen mimetic peptides: the first step was to demonstrate that the typical triple helix structure was conserved in the gas phase for these protonated (PPG)_10_ and (POG)_10_ peptides, under certain experimental conditions (Lalande et al. 2018b). Then, we have irradiated triple helical peptide trimers and hexamers and showed that these non-covalent complexes undergo breakage of the intermolecular H bonds upon the action of ionizing radiation, releasing isolated peptides. In the case of soft X-rays or carbon ions, these peptides further fragment mostly following Gly – Pro backbone bond cleavage (Schwob et al. 2017b; Lalande et al. 2018a; Abdelmouleh et al. 2023).

To know if the propensity of Gly – Pro bond cleavage is sensitive to peptide sequence, we investigated a 26-residue sequence (PGGPPGPKGNSGEPGAPGSKGDTGAK, abbreviated PK26) of human collagen (P02452, CO1A1_HUMAN, UNIPROT). Furthermore, we have also irradiated the same sequence with the three prolines in Yyy position in the XxxYyyGly repeats being substituted by hydroxyprolines: PGGPOGPKGNSGEOGAOGSKGDTGAK (abbreviated PK26-Hyp). The results for photoabsorption in the VUV as well as X-ray photon energy range are consistent with those obtained in the case of the collagen mimetic peptides (see the previous paragraph): ionization and backbone fragmentation of the peptides, the latter increasing in abundance as photon energy rises from 30 to 545 eV (Schwob et al. 2017a). It is noticeable that the Gly – Pro bond close to the N terminus is cleaved with high probability, for two charge states (3 + and 4+) of both peptides (with or without hydroxyprolines).

These results indicate that preferential Gly – Pro bond cleavage is an intrinsic property of collagen mimetic peptides. The latter conclusion is supported by the literature, since the Gly – Pro peptide bonds of (PPG)_10_ and (POG)_10_ peptides were also found to be particularly fragile following UV irradiation in solution (Jariashvili et al. 2012). UV light, especially in the highest energy region UV-C (280–160 nm wavelength), is known to trigger damage to biological molecules through the formation of free radicals, but can also cleave covalent bonds after direct photoabsorption and electronic excitation of biomolecules. For peptides and proteins such as collagen, this is particularly expected around 220 nm, where electrons of the peptide bond strongly absorb UV light. Furthermore, these direct effects are highly relevant for collagen, since it is a major component and its concentration is high in connective tissues. However, UV photoabsorption of isolated collagen-related peptides has not been reported, to the best of our knowledge.

### Present results

**Fig. 4:**
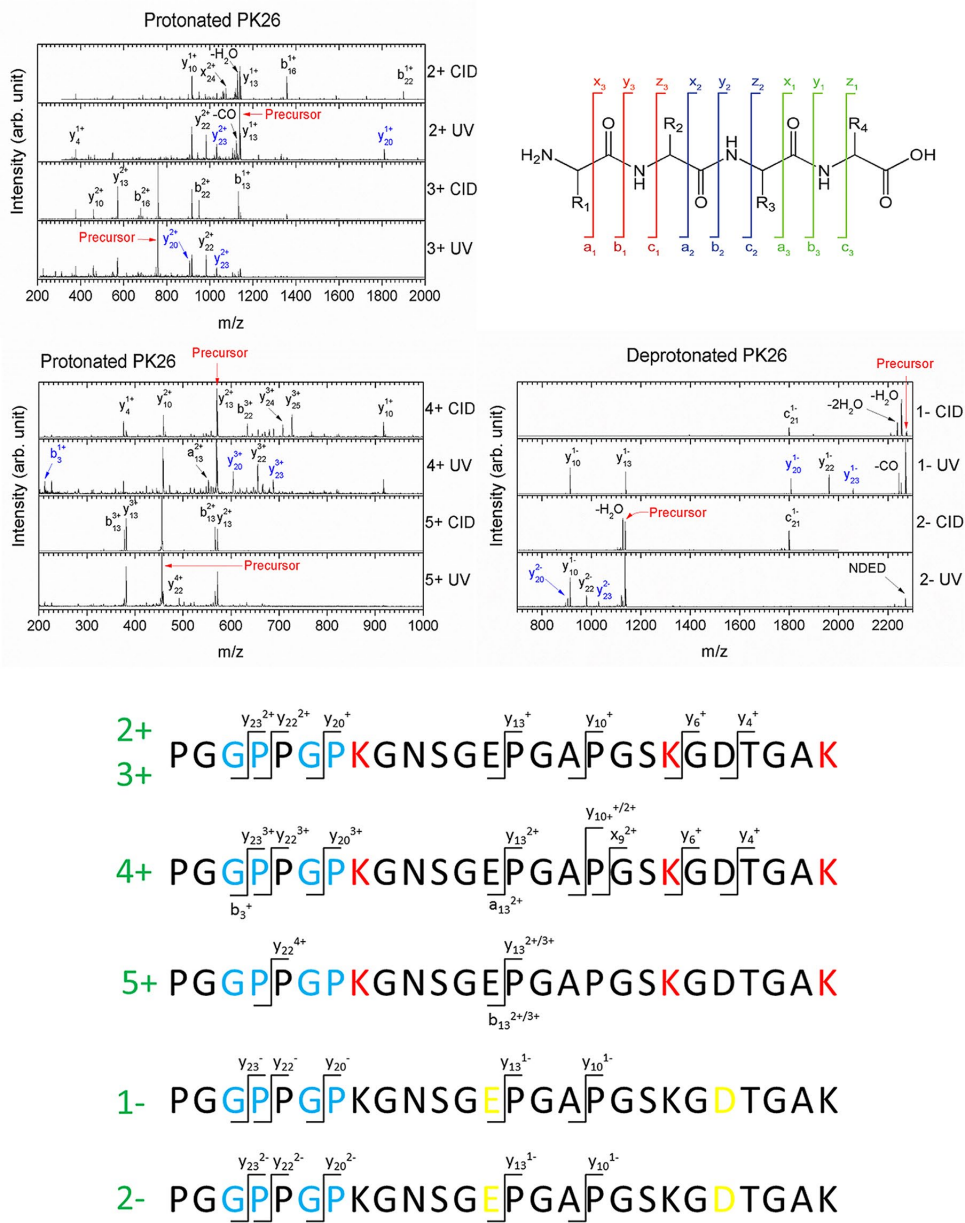
Top: mass spectra of the ionic products of collision-induced dissociation (CID) and UV photoabsorption at 220 nm for the doubly- and triply-protonated PK26 peptide. The usual nomenclature for peptide backbone fragmentation is used and presented on the right for a tetrapeptide with side-chains noted R1 to R4: a, b and c (x, y and z) fragment ions have the charge located on the N-terminal (C-terminal) side after cleavage of the bond crossed by the corresponding line. Fragments from Gly–Pro peptide bond cleavage are depicted in blue and loss of neutral molecules is indicated by a minus sign. Bottom: main backbone fragment ions of the protonated PK26 peptide created after UV photoabsorption, as a function of peptide charge state. Gly–Pro sub-sequences are highlighted in blue, lysine residues in red, acidic residues in yellow

In the following, we present our experimental results regarding UV irradiation of the same peptidic sequence of human collagen as we studied previously (see the previous paragraph). In Fig. 4, the mass spectra containing the cationic products of the 2 + and 3 + charge states of this peptide after UV photoabsorption are shown. Among the most intense peaks, two are assigned to the $\:{y}_{23}$ and $\:{y}_{20}$ fragment ions originating from the cleavage of one or the other of the Gly – Pro peptide bonds. Interestingly, these fragments are hardly formed by collision-induced dissociation (CID, see Fig. 4), a well-known technique used in mass spectrometry to reveal the lowest-energy fragmentation channels in molecular systems following excitation of molecular vibrational modes. Therefore, it indicates that Gly – Pro peptide bond cleavage is due to electronic and not vibrational excitation. Instead, the abundant $\:{y}_{13}$, $\:{y}_{10}$ and $\:{y}_{4}$ fragments are produced by both UV photoabsorption and CID, indicating that cleavage of Glu–Pro, Ala–Pro and Asp–Thr peptide bonds are mostly originating from vibrational excitation. In the case of UV photoabsorption, it thus corresponds to fragmentation in the electronic ground state after internal conversion and redistribution of the excess energy over the vibrational degrees of freedom of the peptide.

The same observations can be made for the 4 + peptide (Fig. 4), although it is worth mentioning that the $\:{y}_{23}^{3+}$ fragment is also formed by CID, and surprisingly, its complementary fragment $\:{b}_{3}^{+}$ is only visible after UV photoabsorption. This $\:{b}_{3}^{+}$ fragment ion is not present for lower charge states of the precursor because of the lowest probability to find a charge close to the N terminus. The 5+ peptide precursor is an exception, since the same behavior stands for CID as well as UV photoabsorption: the peptide breaks almost exclusively in the middle, forming the complementary fragments $\:{y}_{13}$ and $\:{b}_{13}$ (Fig. 4).

Figure 4 summarizes the bonds cleaved by UV photoabsorption. Very similar results were obtained for the hydroxylated peptide PK26-Hyp, showing that hydroxylation of proline does not quench Gly–Pro peptide bond cleavage (Supplementary material, Figure S1). In order to check that this process is not only occurring in protonated species, deprotonated PK26 ions have been studied, and the results are given in Fig. 4. For both singly- and doubly-deprotonated peptides, the Gly–Pro peptide bond is cleaved after UV photoabsorption, forming $\:{y}_{20}$ and $\:{y}_{23}$ fragments whose charge state is consistent with the location of negative charges at acidic residues. Note that Ala–Pro, Pro–Pro as well as Glu–Pro peptide bonds can also be broken. After CID, totally different channels are observed. Another channel specific to UV photoabsorption is non-dissociative electron detachment, detected for the 2- ion but not for the 1- because neutrals are lost in the mass spectrometer. Here again, very similar results were obtained for the PK26-Hyp peptide (see Fig. S1 for supporting information).

Very recently, Kowalewski & Forde have reported very interesting results from UV irradiation of collagen fibrils in the condensed phase (Kowalewski and Forde 2024). By means of SDS-PAGE, they show that as the UV (222 nm wavelength) fluence increases up to 20 J/cm^2^, the bands assigned to intact or cross-linked alpha chains become weaker, and they explain this by chain cleavage. Since no additional band appears, they assume that cleavage is random and/or results in fragments lighter than 40 kDa. These observations and interpretations are perfectly consistent with a preferential Gly-Pro cleavage site in collagen peptides after 220 nm UV photoabsorption, since the Gly-Pro bonds are homogenously distributed in the triple-helical region of type II collagen, and their cleavage can thus produce many small fragments such as the 10–15 amino-acid long peptides that we chose in this study.

Overall, our previous and present experimental results on isolated collagen-related peptides show that Gly–Pro peptide bonds are particularly prone to cleavage following interaction with ionizing radiation such as VUV or X-ray photons, carbon ions, but also with UV radiation, independent of the peptide charge state. Thus, it seems to be a general process triggered by electronic excitation of collagen-related peptides. According to the primary sequence of ECM collagen (P02458, CO2A1_HUMAN Collagen, UNIPROT), 270 proline residues are observed (18% of all amino-acids) and in 47% of the cases, these prolines are N-linked with glycine residues. Each of the other amino-acids N-linked with proline individually represents less than 15%. Thus, the glycine residue is the most likely to be N-linked to a given proline residue in collagen. In the next section, we use these specific Gly-Pro bond to select collagen peptides from the whole sequence of collagen (P02458) and investigate their putative toxicity on chondrocytes.

### Cellular toxicity of selected collagen peptides

A set of collagen peptides to be analyzed was established, according to different parameters. Using the collagen II amino-acid sequence (Uniprot), considering only the cleavage between glycine and proline amino-acids (see the results of the previous section), considering a minimum peptide length of 10 and a maximum peptide length of 15, we propose a list of 46 peptides potentially cleaved following irradiation of collagen in the ECM of cartilage (Supplementary material Table S1). All peptides were synthesized on-demand as powders of over 95% purity (see the Material and Methods section). In an exploratory approach, we tested each of these peptides using the MTT test against T/C-28AC cells in vitro at concentrations ranging from 10^− 4^ to 1 µg/µl. This screening test allows an accurate measurement of cellular metabolic activity, an indicator of cell viability, proliferation and toxicity. It differs from the colony formation assay, which evaluate the ability of a single cell to grow into a colony and to undergo divisions. This screening test is more adapted for this first trial, but the colony formation assay could be tested later on specific peptides.

**Fig. 5:**
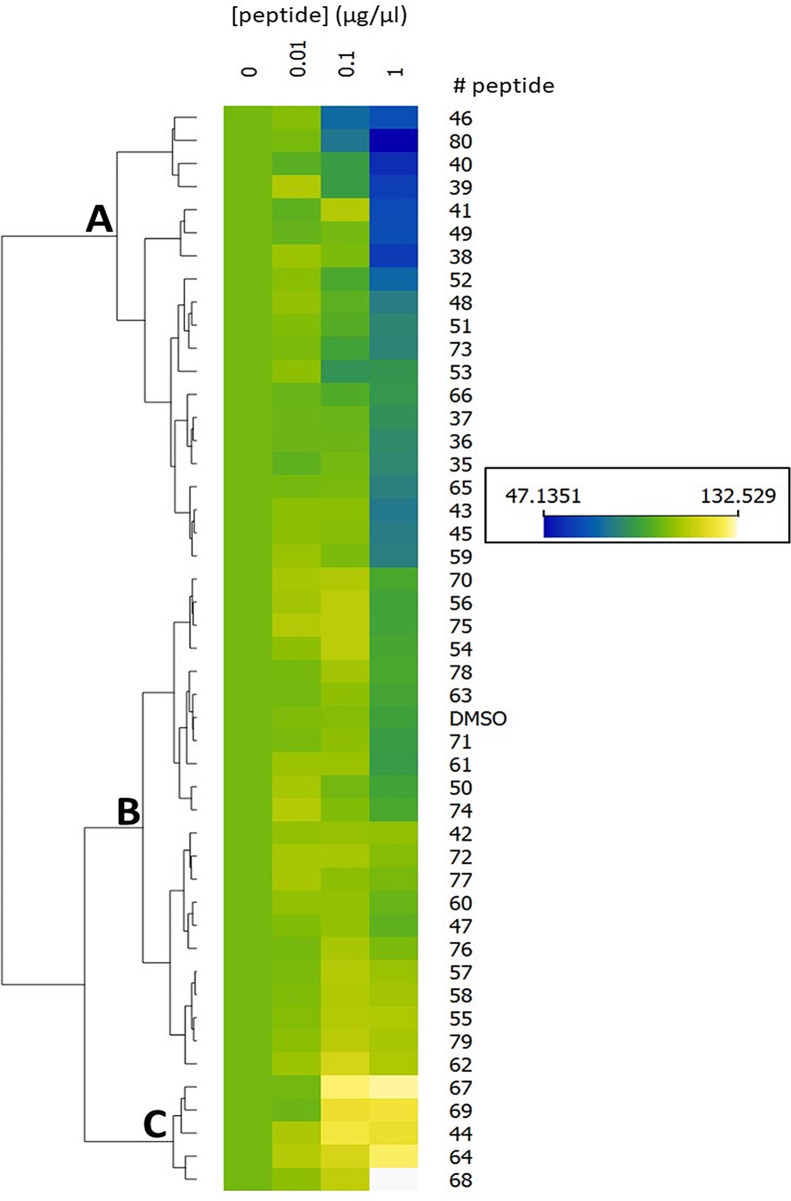
Heatmap displaying the results of a hierarchical classification of collagen peptides according to cell inhibition rate on chondrocytes. Normalized OD values vary from > 115 (highest value, in yellow levels) to < 85 (lowest values, in blue levels), with intermediate values in green levels, (between 85 and 115). Heat map allowed visualizing attribute values in a two-way matrix and clusters data by similarity with hierarchical clustering on Euclidean distances and with average linkage. Done with Orange data mining software V3.36

From the 46 tested peptides, no effects were observed with the lowest concentrations (10^− 4^ and 10^− 3^ µg/µl). Using higher concentrations of peptides (10^− 2^, 10^− 1^, 1 µg/µl), a heatmap and a dendrogram were performed to classify the peptides according to their effects on chondrocytes (Fig. 5). We observed 21 peptides (and the DMSO alone control) with no activity even at the highest concentration (Fig. 5, group B), 20 peptides with a reduction of viability at the highest concentration (up to 53%, see Fig. 5, group A) and 5 peptides with an increase of viability at the highest concentration (up to 33%, see Fig. 5, group C).

**Fig. 6:**
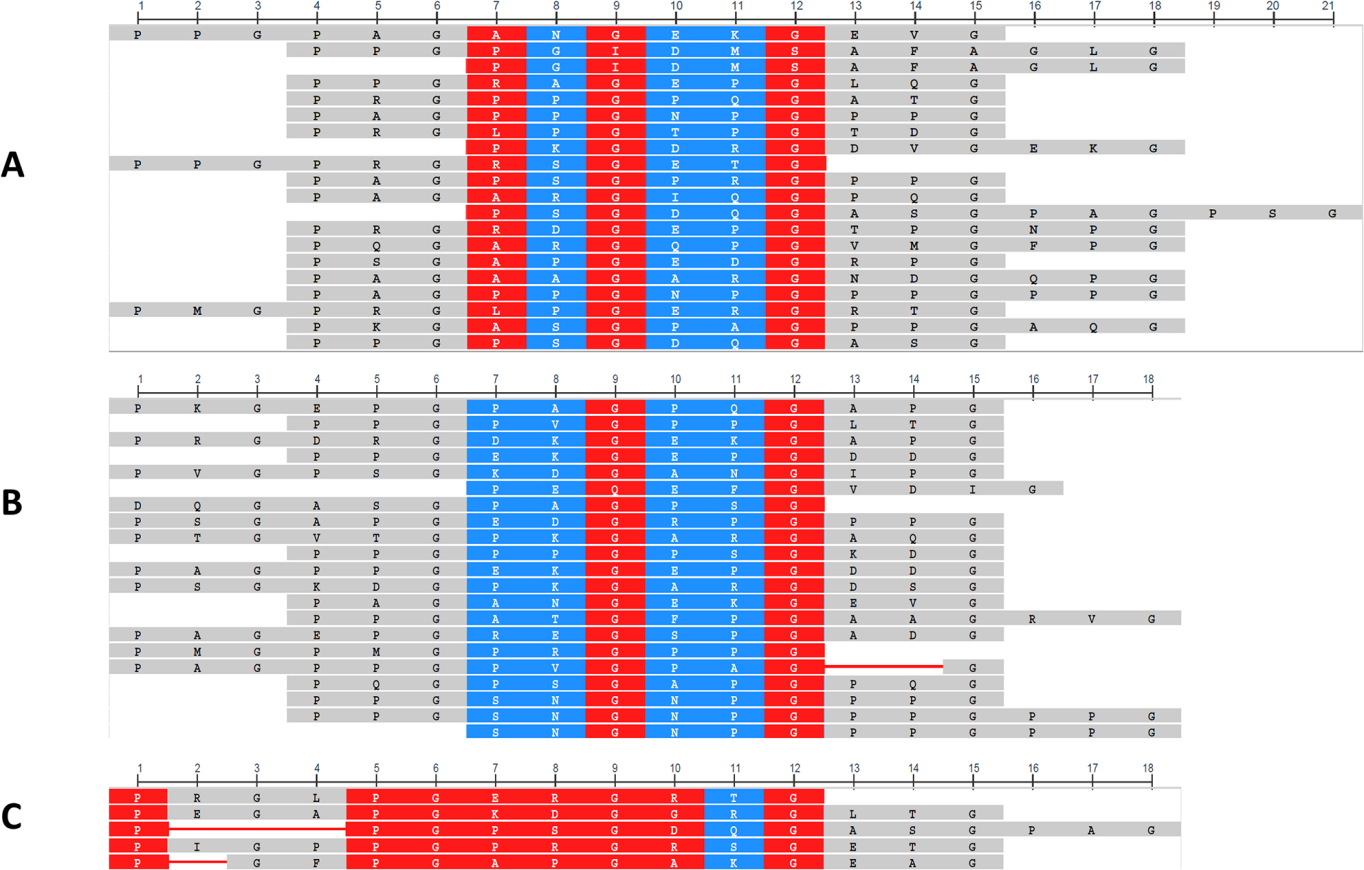
Sequence homologies of collagen peptides according to classification groups (Fig. 5) using the COBALT tool. Only alignment positions with no gaps are colored in the alignment map. Red indicates highly conserved residues and blue indicates lower conservation. See method part for the metric used for peptide alignment

A possible explanation of the observed reduction or increase of viability of chondrocytes in the presence of collagen peptides is the recognition of these peptides by membrane receptors, implying that the peptidic primary and/or secondary structures are important. We thus used the COBALT (Constraint-based Multiple Alignment Tool; U.S. National Library of Medecin, NIH) software, in order to estimate the sequence homology between peptides. COBALT is a multiple sequence alignment tool that finds a collection of pairwise constraints derived from conserved domain database, protein motif database, and sequence similarity. Pairwise constraints are then incorporated into a progressive multiple alignment. This method highlights highly conserved and less conserved amino acid positions based on the relative entropy threshold of the residue (Fig. 6). In addition, the peptides belonging to groups A and C are randomly distributed within the triple helical region of type II collagen (Supplementary material, Figure S2).

First, it is important to keep in mind that glycine is present every three amino acids in collagen, leading to two red columns almost containing only glycine for all peptides. However, we can observe differences in the pattern of the sequence homology between the three groups of peptides (Fig. 6). All 5 peptides with a viability activity present a highly conserved sequence (Fig. 6, group C), with six red and one blue columns in addition to the two “glycine” red columns. Toxic peptides show lower sequence conservation, since only one red and three blue additional columns are observed (Fig. 6, group A). The sequence of peptides with no effect is even less conserved, since only blue additional columns appear (Fig. 6, group B). We have to take these results with care, especially the conserved sequence of viability peptides (group C), since only 5 peptides were aligned in this case, which might modulate positively the sequence homology. However, it might point to specific recognition of collagen matrikines by membrane receptors on chondrocytes. Collagen indeed interacts with at least five groups of cell receptors; among those, integrins are very important. The GFOGER sequence and GPO repeats are specifically recognized by receptors (Knight et al. 2000; Elango et al. 2022). Therefore, we checked in the sequence of α1 type II collagen (see Figure S2) if these sequences were present in the peptides belonging to groups A or C. After translation of collagen, GPP is modified to become GPO after proline hydroxylation in some or all of the collagen chains (Elango et al. 2022). The GFOGER sequence did not belong to one of our tested peptides. Interestingly, GPP is found in 5/20 peptides in group A (a GPPGPP motif is even found in one of the peptides), while only 1/5 peptides in group C contains GPP.

## Conclusion

In this study, we showed for the first time that chondrocytes irradiated in vitro display strong radiation sensitivity as determined with the colony formation assay at low doses of X-rays. This sensitivity seems to be related to bystander factors within the conditioned medium of irradiated cells. The same response was observed when transferring this medium to non-irradiated chondrocytes. Since a little amount of ECM is produced by chondrocytes in the conditions of the present experiments, our hypothesis is that radiation-induced fragments of ECM might contribute to this bystander effect. In order to identify such fragments, we have performed here and in previous work irradiation of isolated collagen-related peptides and found a preferential site of breakage after interaction with UV, VUV and X-ray photons as well as carbon ions: the Gly–Pro peptide bond. Then, we extracted 46 peptides resulting from Gly–Pro cleavages within the type II collagen sequence, and showed that 21 have no effect on chondrocytes, 20 revealed a cytotoxic activity and 5 increased cellular viability in the MTT test. A possible explanation of these effects is specific recognition of these peptides by membrane receptors of chondrocytes, since the involved peptides have more conserved sequences than the 21 with no effect. Taken together, our preliminary results showed that matrikines could be involved in the cellular response to ionizing radiation. As a consequence, cartilage ECM should be preserved as much as possible during therapeutic irradiation in order to keep cellular homeostasis and to prevent cartilage dysfunctions. Further experiments are needed to first clearly establish the link between collagen peptides and bystander effect on chondrocytes in vitro, and then to identify more precisely which sequences are recognized and which receptors are involved in the membrane of chondrocytes: docking experiments and simulations would certainly be useful. A quantification of these specific peptides in the supernatant of irradiated cells would give additional information on the kinetic of production of collagen fragments.

### Electronic supplementary material

Below is the link to the electronic supplementary material.

Supplementary Material 1

## Data Availability

No datasets were generated or analysed during the current study.
