# Supplementary material for: Pilot screening of potential matrikines resulting from collagen breakages through ionizing radiation
Source: Radiat Environ Biophys. 2024 Aug 8;63(3):337–50. doi: 10.1007/s00411-024-01086-z (PMC11341654; doi:10.1007/s00411-024-01086-z)
Supplement: Supplementary file 1 — Supplementary Material 1 [file 411_2024_1086_MOESM1_ESM.pdf]

Supplementary material

From:

Pilot screening of potential matrikines resulting from collagen breakages through ionizing radiation

J. Montanari[1], L. Schwob[2], A. Marie-Brasset[1], C. Vinatier[3], C. Lepleux[1], R. Antoine[4], J. Guicheux[3], J.C. Poully[1*] and F. Chevalier[1*]

[1] UMR6252 CIMAP, CEA - CNRS - ENSICAEN - Université de Caen Normandie, 14000 Caen, France

[2] Deutsches Elektronen-Synchrotron DESY, Notkestr. 85, 22607 Hamburg, Germany

[3] Nantes Université, Oniris, CHU Nantes, INSERM, Regenerative Medicine and Skeleton, RMeS, UMR 1229, F-44000 Nantes, France.

[4] Institut Lumière Matière, University of Lyon, Université Claude Bernard Lyon 1, CNRS, Lyon F-69622, France

* Corresponding authors:

Dr. Jean-Christophe Poully and Dr. François Chevalier

Emails: poully@ganil.fr ; chevalier@ganil.fr

Table S1

List of selected peptide according to the Gly - Pro bond breakage within the collagen II sequence with size between 10 and 15 AA (P02458 - CO2A1_HUMAN ; UniProtKB reviewed, Swiss-Prot)

#	Peptide sequence	Formula	M.W.	Lenght
35	PAGAAGARGNDGQPG	$C_{51}H_{82}N_{20}O_{20}$	1295.32	PG-15
36	PSGAPGEDGRPG	$C_{44}H_{69}N_{15}O_{18}$	1096.11	PG-12
37	PQGARGQPGVMGFPG	$C_{63}H_{98}N_{20}O_{18}S_1$	1455.64	PG-15
38	PRGLPGTPGTDG	$C_{47}H_{77}N_{15}O_{17}$	1124.20	PG-12
39	PPGRAGEPGLQG	$C_{48}H_{78}N_{16}O_{16}$	1135.23	PG-12
40	PGIDMSAFAGLG	$C_{50}H_{78}N_{12}O_{16}S_1$	1135.29	PG-12
41	PRGPPGPQGATG	$C_{46}H_{74}N_{16}O_{15}$	1091.18	PG-12
42	PKGEPGPAGPQGAPG	$C_{57}H_{89}N_{17}O_{19}$	1316.42	PG-15
43	PMGPRGLPGERGRTG	$C_{63}H_{108}N_{24}O_{19}S_1$	1537.75	PG-15
44	PPGPSGDQGASGPAG	$C_{51}H_{78}N_{16}O_{21}$	1251.26	PG-15
45	PKGASGPAGPPGAQG	$C_{53}H_{85}N_{17}O_{18}$	1248.35	PG-15
46	PPGPAGANGEKGEVG	$C_{56}H_{89}N_{17}O_{21}$	1336.41	PG-15
47	PPGPVGPPGLTG	$C_{48}H_{76}N_{12}O_{14}$	1045.19	PG-12
48	PPGPRGRSGETG	$C_{47}H_{78}N_{18}O_{17}$	1167.23	PG-12
49	PAGPPGNPGPPG	$C_{45}H_{67}N_{13}O_{14}$	1014.09	PG-12
50	PRGDRGDKGEKGAPG	$C_{60}H_{101}N_{23}O_{22}$	1496.58	PG-15
51	PAGPSGPRGPPG	$C_{45}H_{71}N_{15}O_{14}$	1046.14	PG-12
52	PKGDRGDVGEKG	$C_{49}H_{83}N_{17}O_{19}$	1214.29	PG-12
53	PSGDQGASGPAGPSG	$C_{49}H_{76}N_{16}O_{22}$	1241.22	PG-15
54	PPGEKGEPGDDG	$C_{47}H_{71}N_{13}O_{21}$	1154.14	PG-12
55	PVGPSGKDGANGIPG	$C_{56}H_{91}N_{17}O_{20}$	1322.42	PG-15
56	PEQEFGVDIG	$C_{48}H_{71}N_{11}O_{18}$	1090.14	PG-10
57	DQGASGPAGPSG	$C_{39}H_{61}N_{13}O_{18}$	999.98	DG-12
58	PSGAPGEDGRPGPPG	$C_{56}H_{86}N_{18}O_{21}$	1347.39	PG-15
59	PPGPSGDQGASG	$C_{41}H_{63}N_{13}O_{18}$	1026.02	PG-12
60	PTGVTGPKGARGAQG	$C_{56}H_{96}N_{20}O_{19}$	1353.48	PG-15
61	PPGPPGPSGKDG	$C_{46}H_{71}N_{13}O_{16}$	1062.13	PG-12
62	PAGPPGEKGEPGDDG	$C_{57}H_{86}N_{16}O_{24}$	1379.39	PG-15
63	PSGKDGPKGARGDSG	$C_{55}H_{92}N_{20}O_{22}$	1385.44	PG-15
64	PIGPPGPRGRSGETG	$C_{60}H_{99}N_{21}O_{20}$	1434.56	PG-15
65	PAGPPGNPGPPGPPG	$C_{57}H_{84}N_{16}O_{17}$	1265.37	PG-15
66	PRGRDGEPGTPGNPG	$C_{59}H_{94}N_{22}O_{22}$	1463.51	PG-15
67	PRGLPGERGRTG	$C_{51}H_{89}N_{21}O_{16}$	1252.38	PG-12
68	PGFPGAPGAKGEAG	$C_{54}H_{81}N_{15}O_{17}$	1212.31	PG-14
69	PEGAPGKDGGRGLTG	$C_{56}H_{93}N_{19}O_{21}$	1368.45	PG-15
70	PAGANGEKGEVG	$C_{44}H_{72}N_{14}O_{18}$	1085.13	PG-12
71	PPGATGFPGAAGRVG	$C_{58}H_{90}N_{18}O_{17}$	1311.45	PG-15
72	PAGEPGREGSPGADG	$C_{54}H_{84}N_{18}O_{23}$	1353.35	PG-15
73	PAGARGIQGPQG	$C_{46}H_{77}N_{17}O_{15}$	1108.21	PG-12
74	PMGPMGPRGPPG	$C_{49}H_{79}N_{15}O_{13}S_2$	1150.37	PG-12
75	PAGPPGPVGPAGG	$C_{46}H_{71}N_{13}O_{14}$	1030.13	PG-13
76	PQGPSGAPGPQG	$C_{44}H_{68}N_{14}O_{16}$	1049.09	PG-12
77	PPGSNGNPGPPG	$C_{44}H_{66}N_{14}O_{16}$	1047.08	PG-12
78	PPGSNGNPGPPGPPG	$C_{56}H_{83}N_{17}O_{19}$	1298.36	PG-15
79	SNGNPGPPGPPG	$C_{44}H_{66}N_{14}O_{16}$	1047.08	SG-12
80	PPGPGIDMSAFAGLG	$C_{62}H_{95}N_{15}O_{19}S_1$	1386.57	PG-15

Figure S1

Top: mass spectra of the ionic products of collision-induced dissociation (CID) and UV photoabsorption at 220 nm for the doubly- and triply-protonated PK26-Hyp peptide. The usual nomenclature for peptide backbone fragmentation is used and presented on the right for a tetrapeptide with side-chains noted R1 to R4: a, b and c (x, y and z) fragment ions have the charge located on the N-terminal (C-terminal) side after cleavage of the bond crossed by the corresponding line. Fragments from Gly–Pro peptide bond cleavage are depicted in blue and loss of neutral molecules is indicated by a minus sign. Bottom: main backbone fragment ions of the protonated PK26-Hyp peptide created after UV photoabsorption, as a function of peptide charge state. O stands for hydroxyproline, Gly–Pro sub-sequences are highlighted in blue, lysine residues in red, acidic residues in yellow.

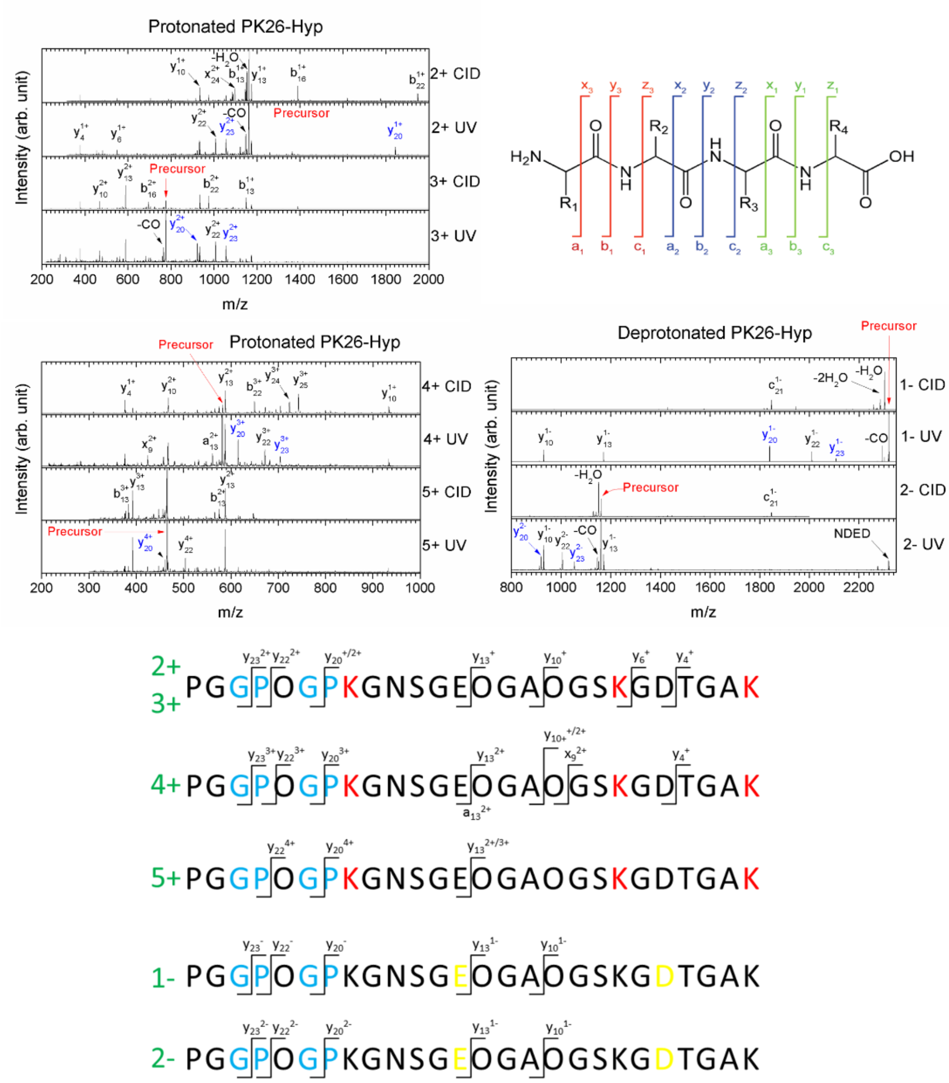

Figures S2

Location of group A peptides within the collagen sequence P02458 - CO2A1_HUMAN

MIRLGAPQTLVLLTLLVAAVLRCQGQDVQEAGSCVQDGQRYNDKDVWKPEPCRICVCDTGTVLCDDIICEDVKDCLSPEIPFGE
CCPICPTDLATASGQPGPKGQKGEPGDIKDIVGPKGPPGPQGPAGEQGPRGDRGDKGEKGAPGPRGRDGEPGTPGNPGPPG
PPGPPGPPGLGGNFAAQMAGGFDEKAGGAQLGVMQGPMGPMGPRGPPGPAGAPGPQGFQGNPGEPGEPGVSGPMGP
RGPPGPPGKPGDDGEAGKPGKAGERGPPGPQGARGFPGTPGLPGVKGHRGYPGLDGAKGEAGAPGVKGESGSPGENGSPG
PMGPRGLPGERGRTGPAGAAGARGNDGQPGPAGPPGPVGPAGGPGFPGAPGAKGEAGPTGARGPEGAQGPRGEPGTPGS
PGPAGASGNPGTDGIPGAKGSAGAPGIAGAPGFPGPRGPPGPQGATGPLGPKGQTGEPGIAGFKGEQGPKGEPGPAGPQGA
PGPAGEEGKRGARGEPGGVGPIGPPGERGAPGNRGFPGQDGLAGPKGAPGERGPSGLAGPKGANGDPGRPGEPGLPGARG
LTGRPGDAGPQGKVGPSGAPGEDGRPGPPGPQGARGQPGVMGFPGPKGANGEPGKAGEKGLPGAPGLRGLPGKDGETGA
AGPPGPAGPAGERGEQGAPGPSGFQGLPGPPGPPGEGGKPGDQGVPGEAGAPGLVGPRGERGFPGERGSPGAQGLQGPR
GLPGTPGTDGPKGASGPAGPPGAQGPPGLQGMPGERGAAGIAGPKGDRGDVGEKGPEGAPGKDGGRGLTGPIGPPGPAGA
NGEKGEVGPPGPAGSAGARGAPGERGETGPPGPAGFAGPPGADGQPGAKGEQGEAGQKGDAGAPGPQGPSGAPGPQGPT
GVTGPKGARGAQGPPGATGFPGAAGRVGPPGSNGNPGPPGPPGPSGKDGPKGARGDSGPPGRAGEPGLQGPAGPPGEKG
EPGDDGPSGAEGPPGPQGLAGQRGIVGLPGQRGERGFPGLPGPSGEPGKQGAPGASGDRGPPGPVGPPGLTGPAGEPGREG
SPGADGPPGRDGAAGVKGDRGETGAVGAPGAPGPPGSPGPAGPTGKQGDRGEAGAQGPMGPSGPAGARGIQGPQGPRG
DKGEAGEPGERGLKGHRGFTGLQGLPGPPGPSGDQGASGPAGPSGPRGPPGPVGPSGKDGANGIPGPIGPPGPRGRSGETG
PAGPPGNPGPPGPPGPPGPGIDMSAFAGLGPREKGPDPLQYMRADQAAGGLRQHDAEVDATLKSLNNQIESIRSPEGSRKNP
ARTCRDLKLCHPEWKSGDYWIDPNQGCTLDAMKVFCNMETGETCVYPNPANVPKKNWWSSKSKEKKHIWFGETINGGFHFS
YGDDNLAPNTANVQMTFLRLLSTEGSQNITYHCKNSIAYLDEAAGNLKKALLIQGSNDVEIRAEGNSRFTYTALKDGCTKHTGK
WGKTVIEYRSQKTSRLPIIDIAPMDIGGPEQEFGVDIGPVCFL

46 PPGPAGANGEKGEVG
80 PPGPGIDMSAFAGLG
40 PGIDMSAFAGLG
39 PPGRAGEPGLQG
41 PRGPPGPQGATG
49 PAGPPGNPGPPG
38 PRGLPGTPGTDG
52 PKGDRGDVGEKG
48 PPGPRGRSGETG
51 PAGPSGPRGPPG
73 PAGARGIQGPQG
53 PSGDQGASGPAGPSG
66 PRGRDGEPGTPGNPG
35 PAGAAGARGNDGQPG
36 PSGAPGEDGRPG
37 PQGARGQPGVMGFPG
65 PAGPPGNPGPPGPPG
43 PMGPRGLPGERGRTG
45 PKGASGPAGPPGAQG
59 PPGPSGDQGASG

Location of group C peptides within the collagen sequence P02458 - CO2A1_HUMAN

MIRLGAPQTLVLLTLLVAAVLRCQGQDVQEAGSCVQDGQRYNDKDVWKPEPCRICVCDTGTVLCDDIICEDVKDCLSPEIPFGE
CCPICPTDLATASGQPGPKGQKGEPGDIKDIVGPKGPPGPQGPAGEQGPRGDRGDKGEKGAPGPRGRDGEPGTPGNPGPPG
PPGPPGPPGLGGNFAAQMAGGFDEKAGGAQLGVMQGPMGPMGPRGPPGPAGAPGPQGFQGNPGEPGEPGVSGPMGP
RGPPGPPGKPGDDGEAGKPGKAGERGPPGPQGARGFPGTPGLPGVKGHRGYPGLDGAKGEAGAPGVKGESGSPGENGSPG
PMGPRGLPGERGRTGPAGAAGARGNDGQPGPAGPPGPVGPAGGPGFPGAPGAKGEAGPTGARGPEGAQGPRGEPGTPGS
PGPAGASGNPGTDGIPGAKGSAGAPGIAGAPGFPGPRGPPGPQGATGPLGPKGQTGEPGIAGFKGEQGPKGEPGPAGPQGA
PGPAGEEGKRGARGEPGGVGPIGPPGERGAPGNRGFPGQDGLAGPKGAPGERGPSGLAGPKGANGDPGRPGEPGLPGARG
LTGRPGDAGPQGKVGPSGAPGEDGRPGPPGPQGARGQPGVMGFPGPKGANGEPGKAGEKGLPGAPGLRGLPGKDGETGA
AGPPGPAGPAGERGEQGAPGPSGFQGLPGPPGPPGEGGKPGDQGVPGEAGAPGLVGPRGERGFPGERGSPGAQGLQGPR
GLPGTPGTDGPKGASGPAGPPGAQGPPGLQGMPGERGAAGIAGPKGDRGDVGEKGPEGAPGKDGGRGLTGPIGPPGPAGA
NGEKGEVGPPGPAGSAGARGAPGERGETGPPGPAGFAGPPGADGQPGAKGEQGEAGQKGDAGAPGPQGPSGAPGPQGPT
GVTGPKGARGAQGPPGATGFPGAAGRVGPPGSNGNPGPPGPPGPSGKDGPKGARGDSGPPGRAGEPGLQGPAGPPGEKG
EPGDDGPSGAEGPPGPQGLAGQRGIVGLPGQRGERGFPGLPGPSGEPGKQGAPGASGDRGPPGPVGPPGLTGPAGEPGREG
SPGADGPPGRDGAAGVKGDRGETGAVGAPGAPGPPGSPGPAGPTGKQGDRGEAGAQGPMGPSGPAGARGIQGPQGPRG
DKGEAGEPGERGLKGHRGFTGLQGLPGPPGPSGDQGASGPAGPSGPRGPPGPVGPSGKDGANGIPGPIGPPGPRGRSGETG
PAGPPGNPGPPGPPGPPGPGIDMSAFAGLGPREKGPDPLQYMRADQAAGGLRQHDAEVDATLKSLNNQIESIRSPEGSRKNP
ARTCRDLKLCHPEWKSGDYWIDPNQGCTLDAMKVFCNMETGETCVYPNPANVPKKNWWSSKSKEKKHIWFGETINGGFHFS
YGDDNLAPNTANVQMTFLRLLSTEGSQNITYHCKNSIAYLDEAAGNLKKALLIQGSNDVEIRAEGNSRFTYTALKDGCTKHTGK
WGKTVIEYRSQKTSRLPIIDIAPMDIGGPEQEFGVDIGPVCFL

67	PRGLPGERGRTG
69	PEGAPGKDGGRGLTG
44	PPGPSGDQGASGPAG
64	PIGPPGPRGRSGETG
68	PGFPGAPGAKGEAG

Domains of Collagen alpha-1(II) chain (UNIPROT P02458 - CO2A1_HUMAN)

MIRLGAPQTLVLLTLLVAAVLRCQGQDVQEAGSCVQDGQRYNDKDVWKPEPCRICVCDTGTVLCDDIICEDVKDCLSPEIPFGE
CCPICPTDLATASGQPGPKGQKGEPGDIKDIVGPKGPPGPQGPAGEQGPRGDRGDKGEKGAPGPRGRDGEPGTPGNPGPPG
PPGPPGPPGLGGNFAAQMAGGFDEKAGGAQLGVMQGPMGPMGPRGPPGPAGAPGPQGFQGNPGEPGEPGVSGPMGP
RGPPGPPGKPGDDGEAGKPGKAGERGPPGPQGARGFPGTPGLPGVKGHRGYPGLDGAKGEAGAPGVKGESGSPGENGSPG
PMGPRGLPGERGRTGPAGAAGARGNDGQPGPAGPPGPVGPAGGPGFPGAPGAKGEAGPTGARGPEGAQGPRGEPGTPGS
PGPAGASGNPGTDGIPGAKGSAGAPGIAGAPGFPGPRGPPGPQGATGPLGPKGQTGEPGIAGFKGEQGPKGEPGPAGPQGA
PGPAGEEGKRGARGEPGGVGPIGPPGERGAPGNRGFPGQDGLAGPKGAPGERGPSGLAGPKGANGDPGRPGEPGLPGARG
LTGRPGDAGPQGKVGPSGAPGEDGRPGPPGPQGARGQPGVMGFPGPKGANGEPGKAGEKGLPGAPGLRGLPGKDGETGA
AGPPGPAGPAGERGEQGAPGPSGFQGLPGPPGPPGEGGKPGDQGVPGEAGAPGLVGPRGERGFPGERGSPGAQGLQGPR
GLPGTPGTDGPKGASGPAGPPGAQGPPGLQGMPGERGAAGIAGPKGDRGDVGEKGPEGAPGKDGGRGLTGPIGPPGPAGA
NGEKGEVGPPGPAGSAGARGAPGERGETGPPGPAGFAGPPGADGQPGAKGEQGEAGQKGDAGAPGPQGPSGAPGPQGPT
GVTGPKGARGAQGPPGATGFPGAAGRVGPPGSNGNPGPPGPPGPSGKDGPKGARGDSGPPGRAGEPGLQGPAGPPGEKG
EPGDDGPSGAEGPPGPQGLAGQRGIVGLPGQRGERGFPGLPGPSGEPGKQGAPGASGDRGPPGPVGPPGLTGPAGEPGREG
SPGADGPPGRDGAAGVKGDRGETGAVGAPGAPGPPGSPGPAGPTGKQGDRGEAGAQGPMGPSGPAGARGIQGPQGPRG
DKGEAGEPGERGLKGHRGFTGLQGLPGPPGPSGDQGASGPAGPSGPRGPPGPVGPSGKDGANGIPGPIGPPGPRGRSGETG
PAGPPGNPGPPGPPGPPGPGIDMSAFAGLGPREKGPDPLQYMRADQAAGGLRQHDAEVDATLKSLNNQIESIRSPEGSRKNP
ARTCRDLKLCHPEWKSGDYWIDPNQGCTLDAMKVFCNMETGETCVYPNPANVPKKNWWSSKSKEKKHIWFGETINGGFHFS
YGDDNLAPNTANVQMTFLRLLSTEGSQNITYHCKNSIAYLDEAAGNLKKALLIQGSNDVEIRAEGNSRFTYTALKDGCTKHTGK
WGKTVIEYRSQKTSRLPIIDIAPMDIGGPEQEFGVDIGPVCFL

Fibrillar collagen NC1

Triple-helical region

GFPGER (GFOGER after proline hydroxylation) : Collagen-binding A-domains of Integrins alpha1 beta 1 and alpha 2 beta1
